# Antihypertensive Drug Use and the Risk of Depression: A Systematic Review and Network Meta-analysis

**DOI:** 10.3389/fphar.2021.777987

**Published:** 2021-11-08

**Authors:** Ying Li, Yuanming Fan, Yangyang Sun, Raphael N. Alolga, Pingxi Xiao, Gaoxiang Ma

**Affiliations:** ^1^ State Key Laboratory of Natural Medicines, School of Traditional Chinese Pharmacy, China Pharmaceutical University, Nanjing, China; ^2^ Department of Cardiology, Sir Run Run Hospital, Nanjing Medical University, Nanjing, China.

**Keywords:** antihypertensive medication, depression, meta-analysis, network meta-analysis, review

## Abstract

**Background:** Although numerous cohort studies have reported an association between antihypertensives use and depression, the exact effect of antihypertensives on depression remains unclear.

**Objective:** To clarify the association between antihypertensives use and risk of depression.

**Methods:** We retrieved relevant literature using PubMed database until August 30, 2021. Four main classes of antihypertensives, thus, angiotensin antagonists, beta blockers, calcium channel blockers and diuretics were studied. The incidence of depression was pooled based on a single drug category. Network meta-analyses were conducted to comprehensively assess the effects of the four classes of antihypertensives on the risk of depression.

**Results:** A total of nine out of 9,557 studies involving 414,873 subjects were retrieved. The pooled results showed a positive association between the use of calcium channel blockers and symptoms of depression [odds ratio (OR): 1.09, 95% confidence interval (CI):1.06–1.13], while use of the angiotensin antagonists, beta blockers and diuretics was not associated with risk of depression. Subgroup analysis suggested a significant relationship between beta blockers usage and risk of depression in cohort studies (OR:1.21, 95% CI: 1.16–1.26). The results of network meta-analysis indicated that all other three classes of drugs increased the risk of depression: angiotensin antagonists (OR: 1.30, 95% CI: 1.04–1.63), beta blockers (OR: 1.53, 95% CI: 1.22–1.91), and calcium channel blockers (OR: 1.40, 95% CI: 1.12–1.75), compared with diuretics.

**Conclusion:** In conclusion, our results indicate that the use of angiotensin antagonists, beta blockers and calcium channel blockers are potential risk factors of depression.

## Introduction

Depression is the most common psychiatric disorder globally (Raič, 2017). Major depression is estimated to rank as the leading cause of global disease burden by 2030 (Malhi and Mann, 2018). The World Mental Health Survey across 17 countries found that on average, approximately 1 in 20 people have experienced depression (Li et al., 2015). A large number of studies focusing on the etiology of depression have been performed (Yaniv et al., 2010; Foster and McVey Neufeld, 2013; Otte et al., 2016; Cathomas et al., 2019; Beurel et al., 2020), however, the risk factors of depression remain unclear.

Several large-sized sample cohort studies have hinted on the fact that hypertension may accompany a high incidence of depression and possibly affect its treatment and prognosis (Zhang et al., 2018). Use of antihypertensives, primarily the angiotensin-converting enzyme inhibitors (ACEI), angiotensin receptor blockers (ARB), beta blockers, calcium channel blockers and diuretics, has been implicated in depression (James et al., 20142014; Cruickshank, 2017; Agustini et al., 2020; Kessing et al., 2020).

Despite the numerous studies on the subject matter, there is still lack of clarity on whether or not antihypertensives use increases the risk of depression. Some studies have implicated antihypertensives use in depression (Nasr et al., 2011; Simonson et al., 2011; Agustini et al., 2020; Kessing et al., 2020), while others found no association between the two (Feng et al., 2008; Agustini et al., 2020). Given the large number of hypertensive subjects and the severity of depression, it is therefore justifiable to clarify the exact effect of antihypertensives on depression.

Hence, we have summarized studies to uncover the associations between antihypertensive drugs use and depression with the aim of benefitting the management of depression.

## Methods

### Search Strategy and Selection Criteria

We searched the PubMed database using and expanding the MeSH terms “antihypertensive agents” and “depression” until August 30, 2021. The full search terms were illustrated in Supplementary Appendix S1. Literature retrieval was limited to human studies published in English. Preferred Reporting Items for Systematic Reviews and Meta-analyses (PRISMA) guidelines for systematic reviews were followed and fulfilled (Supplementary Appendix S2) (Page et al., 2021). Publication that simultaneously fulfilled the following criteria were included in our study: 1) control groups were users of other classes of medication and/or nonmedicated participants; 2) studies that used a validated method to assess depression or symptoms of depression, and the measure of depression was used as a categorical variable rather than a continuous variable; and 3) studies with sample size of more than 100, so as to avoid selection bias. The selection of relevant literature was independently conducted by two researchers, and disagreements were resolved by consulting a third reviewer.

### Data Extraction and Quality Assessment

For all eligible studies, two researchers independently extracted the following data: the first author, PMID, the year of publication, country, study type, sample age range, percentage of males, the methods used to define depression, odds ratio (OR) or relative risk (RR) and the corresponding 95% CI, as well as control variables for adjustment. For the analysis model used in the studies, we gave priority to multivariate analysis or adjusted OR/RR values over univariate analysis or crude results. If the studies failed to report OR/RR, the raw data were reviewed to determine whether the OR/RR could be calculated. Different antihypertensive medication stratifications were treated as several independent results with corresponding populations separately. If there was stratification by the number of antihypertensive agents, the effect estimates were regarded as independent results separately.

The quality evaluation was assessed by two researchers independently. The Agency for Healthcare Research and Quality (AHRQ) was used to evaluate cross-sectional studies and the Newcastle-Ottawa Scale (NOS) was used for cohort studies (Rostom et al., 2004; Wells et al., 2014). The AHRQ comprised 11 items, with a scale ranging from 0 to 11 (Supplementary Appendix S3). Scores of 8–11 were regarded as high quality, and scores of 4–6 were regarded as moderate quality. For the NOS, an overall quality score ranged from 0 to 9 stars (Supplementary Appendix S4). When a study obtained more than six scores, it was regarded as high quality. When a study obtained scores of 4–6, it was regarded as moderate quality. According to the AHRQ and NOS, all studies involved were of high and moderate quality (Supplementary Table S1).

### Statistical Analysis

In meta-analysis, the degree of heterogeneity was assessed using the *I* ^2^ statistic (Higgins et al., 2003). An *I* ^2^ value < 50% was considered an acceptable level of heterogeneity and we used the fixed effects meta-analysis model to pool data; otherwise, the random-effects model was used. The combined effect estimates were shown as pooled ORs with 95% confidence intervals (CIs) and *p* values. The antihypertensive drugs were divided into four groups: angiotensin antagonists, beta blockers, calcium channel blockers and diuretics. ACEI and ARB were grouped together as angiotensin antagonists. The incidence of depression was pooled based on the single drug category for all subsequent analysis. Sensitivity analysis was conducted by omitting one study each time. Publication bias was investigated using funnel plots. Subgroup analysis was performed according to the type of study (cohort/cross-sectional). To investigate the impact of confounding factors in the control group, subgroup analysis was also conducted by dividing the control group into two subgroups, with the first subgroup not taking antihypertensive medication (NoAntiHTN) as a control and the second subgroup receiving other classes of antihypertensive drugs (AntiHTN) as a reference.

The network meta-analysis was performed using the frequentist approach in the package “netmeta” (version 1.5–0) in R (version 4.1.0; https://www.r-project.org/). The random-effects model was conducted in network meta-analysis to minimize the influence of heterogeneity. Treatments were ranked using P-score which were based solely on the point estimates and standard errors of the network estimates (Rücker and Schwarzer, 2015). Heterogeneity between studies was assessed using Cochran Q-statistics, and *p* value < 0.05 was considered suggestive of significant heterogeneity. Moreover, a net heat plot was employed to locate inconsistency in network meta-analysis. A comparison-adjusted funnel plot was used to assess publication bias. All statistical analyses were conducted using R version 4.1.0, with a 0.05 significance level.

## Results

### Study Selection and Characteristics

**FIGURE 1:**
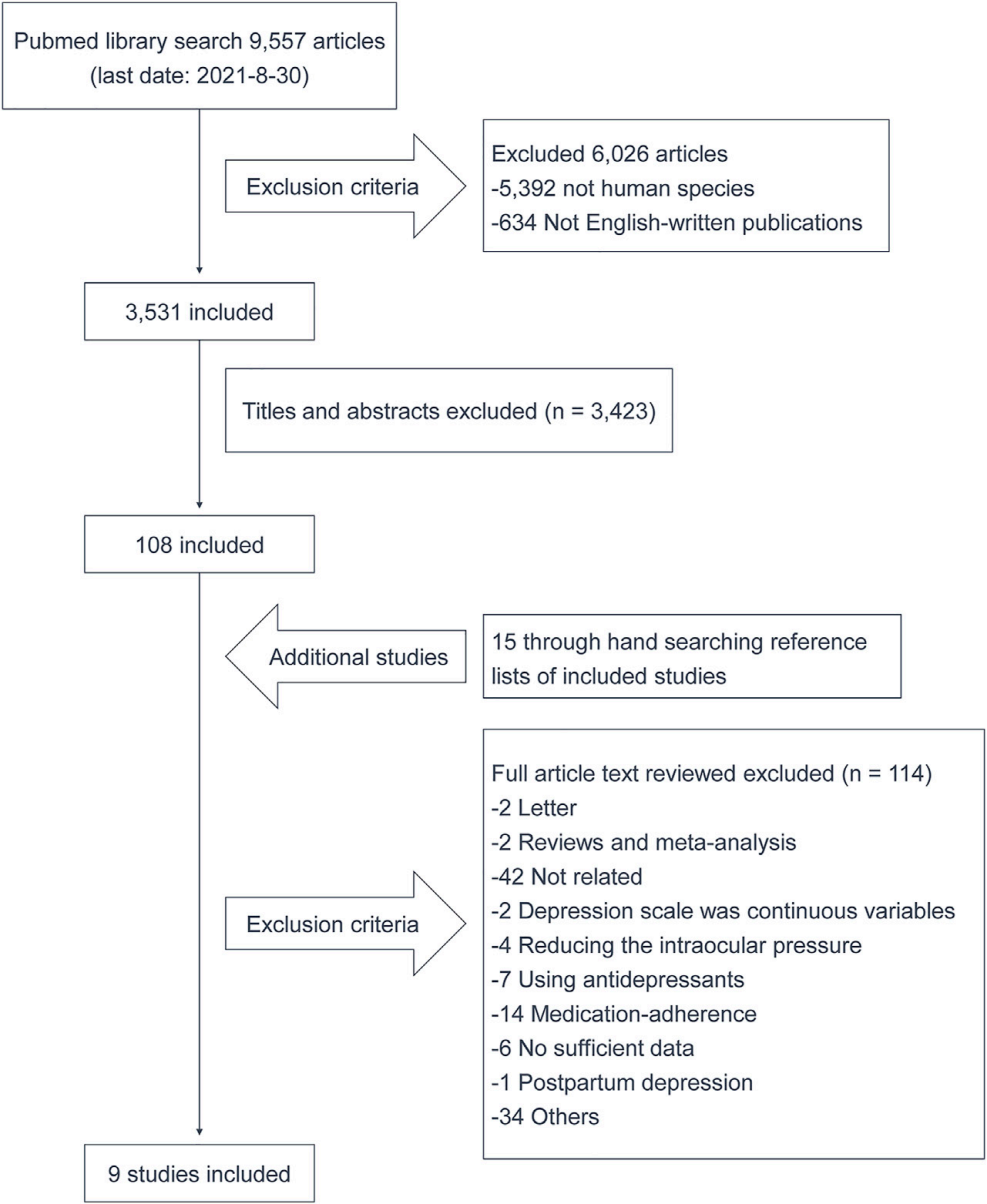
Flow chart of published studies included and excluded in this study.

The workflow of study selection is illustrated in Figure 1. In brief, a total of 9,557 publications were retrieved from the PubMed database, and 9 studies consisting 6 cross-sectional studies and 3 cohort studies were finally involved in this study (Gerstman et al., 1996; Feng et al., 2008; Simonson et al., 2011; Johansen et al., 2012; Michal et al., 2013; Ringoir et al., 2014; Boal et al., 2016; Cao et al., 2019; Agustini et al., 2020).

Characteristics of selected publications are presented in Table 1. Overall 414,873 individuals were included in the final analysis. The sample sizes ranged from 573 to 181,709 individuals. Subjects were recruited from a total of 10 countries, including Australia, China, Germany, Netherlands, Norway, Singapore, the United Kingdom and the United States. Of note, the methods used to assess depression or symptoms of depression varied across the nine studies. The two main diagnostic classification systems were Diagnostic and Statistical Manual of Mental Disorders and International Classification of Diseases (Malhi and Mann, 2018). In all nine studies, three studies identified depression based on these two criteria (Gerstman et al., 1996; Simonson et al., 2011; Boal et al., 2016).

**TABLE 1:** Characteristics of included studies.

Author	Type of medication	Year	Country	Effect measures	OR (95%CI)	Age range (years)	Male (%)	Sample	Definition of depression
Agustini *et al*	ACEI	2020	Australia, United States	OR	1.08 (0.95–1.23)	≥65	46.2	14,195	CES-D
Agustini *et al*	ARB	2020		OR	0.99 (0.89–1.12)		38.6		
Agustini *et al*	BB	2020		OR	1.37 (1.16–1.60)		30.8		
Agustini *et al*	CCB	2020		OR	1.05 (0.92–1.19)		42.2		
Boal *et al*	AA	2016	United Kingdom	OR	0.54 (0.30–0.97)	40–80	56	144,066	ICD-9 and ICD-10
Boal *et al*	BB	2016		OR	1.06 (0.70–1.60)		45.1		
Boal *et al*	CCB	2016		OR	1.17 (0.68–2.01)		48.5		
Boal *et al*	TZ	2016		OR	0.90 (0.42–1.90)		28.5		
Cao *et al*	ACEI	2019	China	RR	1.24 (1.18–1.31)	≥18	50.9	181,709	antidepressant prescription
Cao *et al*	BB	2019		RR	1.21 (1.16–1.26)		44.3		
Cao *et al*	CCB	2019		RR	1.10 (1.06–1.14)		47.3		
Cao *et al*	DIU	2019		RR	1.02 (0.94–1.09)		51.3		
Feng *et al*	ACEI	2008	Singapore	OR	1.11 (0.73–1.68)	≥55	NR	2,804	GDS
Feng *et al*	BB	2008		OR	0.90 (0.66–1.21)				
Feng *et al*	CCB	2008		OR	0.91 (0.65–1.29)				
Feng *et al*	DIU	2008		OR	1.07 (0.68–1.67)				
Gerstman *et al*	BB	1996	United States	RR	0.8 (0.3–1.9)	NR	38.3	3,782	DSM-III-R
Johansen *et al*	ACEI	2012	Norway	OR	0.54 (0.28–1.08)	≥20	NR	55,472	HADS
Johansen *et al*	BB	2012		OR	1.20 (0.78–1.83)				
Johansen *et al*	CCB	2012		OR	1.04 (0.70–1.53)				
Michal *el al*	BB	2013	Germany	OR	1.45 (1.06–1.98)	35–74	NR	5,000	PHQ-9
Michal *et al*	ACEI	2013		OR	1.23 (0.91–1.66)				
Michal *et al*	CCB	2013		OR	0.81 (0.49–1.33)				
Michal *et al*	DIU	2013		OR	1.09 (0.66–1.78)				
Ringoir *et al*	Lipophilic BB	2014	Netherlands	OR	1.60 (1.08–2.36)	60–85	43	573	PHQ-9
Simonson *et al*	BB	2010	United States	OR	0.76 (0.63–0.90)	NR	NR	7,272	ICD-9-CM

ACEI, angiotensin-converting enzyme inhibitors; ARB, angiotensin receptor blockers; CCB, calcium channel blockers; BB, beta blockers; DIU, diuretics; TZ, thiazide diuretics; AA, angiotensin antagonists; OR, odds ratio; CI, confidence interval; RR, relative risk; NR, not reported; CES-D, Center for Epidemiological Studies Depression; ICD, International Classification of Diseases; GDS, Geriatric Depression Scale; DSM, Diagnostic and Statistical Manual of Mental Disorders; HADS, Hospital Anxiety and Depression rating Scale; PHQ-9, Patient Health Questionnaire 9.

### Pooled Results of the Meta-analysis

**FIGURE 2:**
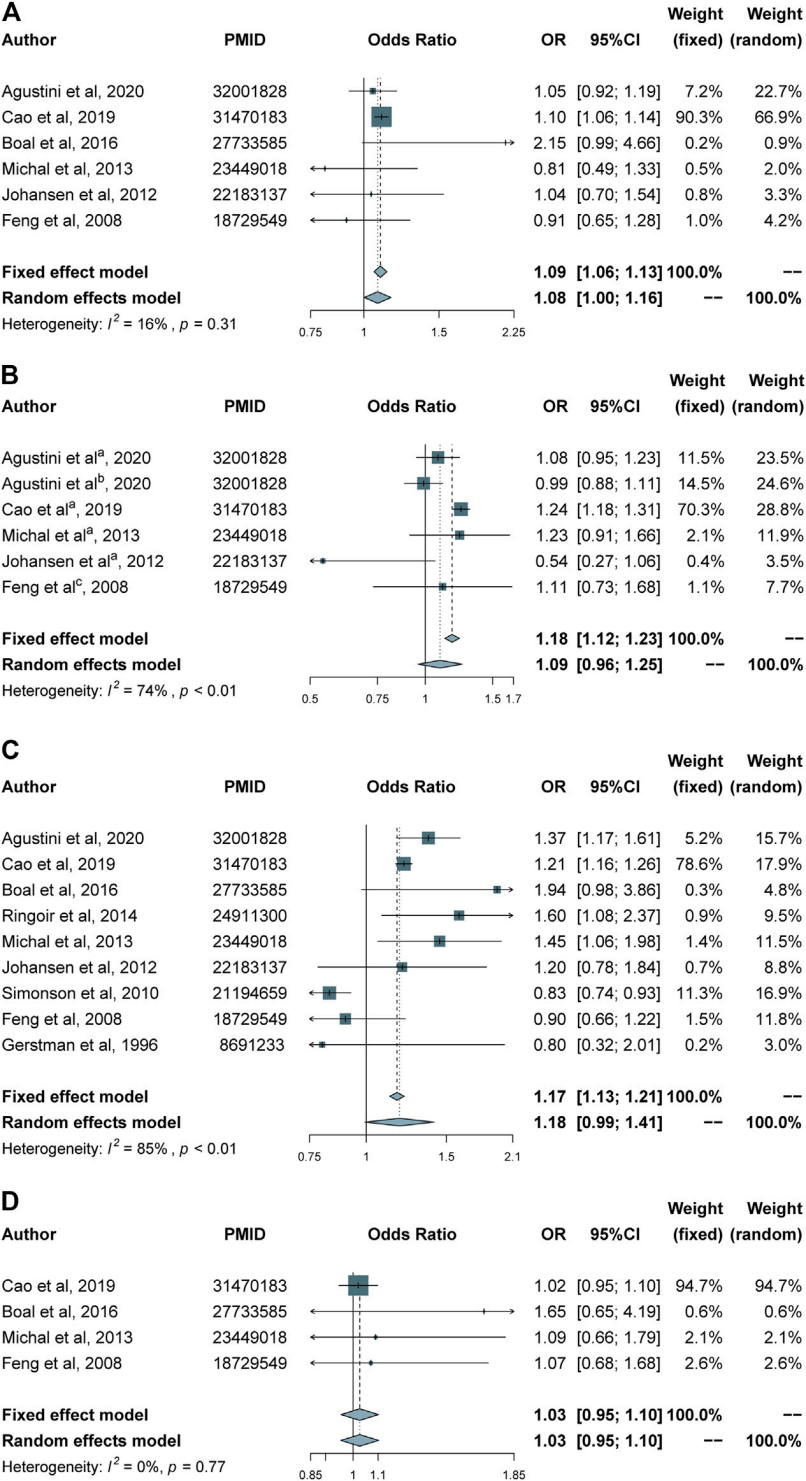
Forest plots for different classes of antihypertensives and risk of depression. **(A)** Calcium channel blockers group. **(B)** Angiotensin antagonists group. **(C)** Beta blockers group. **(D)** Diuretics group. ^**(a–c)**^ indicate angiotensin-converting enzyme inhibitors, angiotensin receptor blockers and angiotensin antagonists, respectively.

Calcium channel blockers use was significantly associated with an increased risk of depression (OR 1.09, 95% CI 1.06–1.13; Figure 2A). Other antihypertensive medications, including angiotensin antagonists, beta blockers and diuretics, showed no significant associations with the incidence of depression or symptoms of depression (Figures 2B–D). The pooled ORs and 95% CI of depression were 1.09 (95% CI 0.96–1.25) for angiotensin antagonists, 1.18 (95% CI 0.99–1.41) for beta blockers and 1.03 (95% CI 0.95–1.10) for diuretics, respectively.

### Heterogeneity and Publication Bias

Given the obvious heterogeneity shown in the forest plots, the sensitivity analysis was conducted by omitting one study at a time (Supplementary Figure S1). In line with the results earlier mentioned, associations between angiotensin antagonists and diuretics use and depression were little, and the result was not excessively influenced by any single study (Supplementary Figures S1A,B). After removing the study by Simonson *et al.*, a significant association between use of beta blockers and depression was observed (pooled OR: 1.26; 95% CI, 1.12–1.42) with the heterogeneity (*I* ^2^ value) decreasing from 85 to 40% (Supplementary Figure S1C). When the study by Cao *et al.* was excluded, no association was found between calcium channel blockers usage and depression (Supplementary Figure S1D).

To evaluate publication bias, funnel plots were constructed. No publication bias was found in the beta blockers group (Supplementary Figure S2A), while apparent biases were found in the other three groups (Supplementary Figures S2B–D).

### Subgroup Analysis

#### Type of Control Group

**FIGURE 3:**
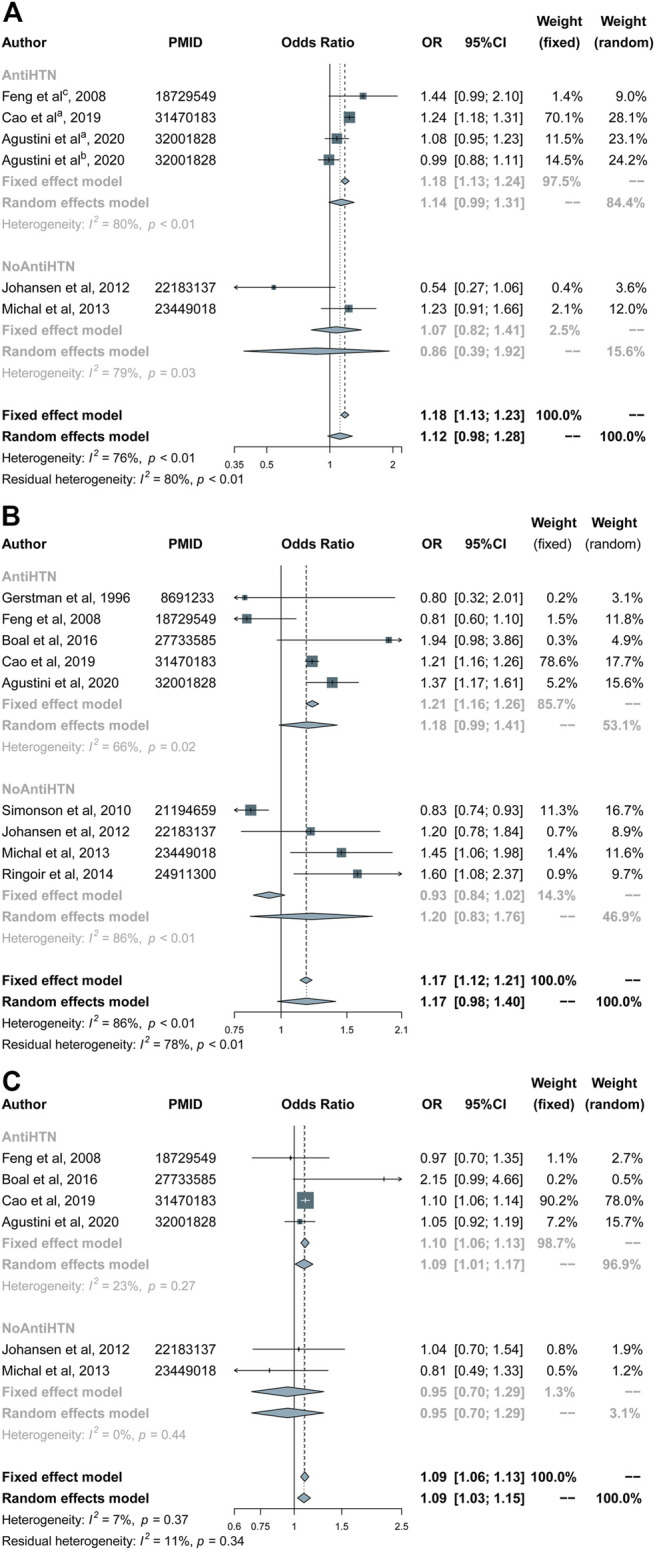
Forest plots of subgroup analysis according to the type of control group (NoAntiHTN/AntiHTN) in **(A)** angiotensin antagonists, **(B)** beta blockers and **(C)** calcium channel blockers group. ^**(a–c)**^ indicate angiotensin-converting enzyme inhibitors, angiotensin receptor blockers and angiotensin antagonists, respectively.

Given the heterogeneity of the controls, subgroup analysis was performed via dividing the controls into two subgroups, NoAntiHTN and AntiHTN, to investigate the impact of antihypertensive drugs use. The subgroup analysis of AntiHTN control group indicated use of antihypertensives increased risk of depression: angiotensin antagonists (OR 1.14, 95% CI 0.99–1.31), beta blockers (OR 1.18, 95% CI 0.99–1.41), and calcium channel blockers (OR 1.10, 95% CI 1.06–1.13) (Figure 3). However, the subgroup analysis of NoAntiHTN control group found no relationship between any class of antihypertensive drugs and risk of depression: angiotensin antagonists (OR 0.86, 95% CI 0.39–1.92), beta blockers (OR 1.20, 95% CI 0.83–1.76), and calcium channel blockers (OR 0.95, 95% CI 0.70–1.29) (Figure 3).

#### Type of Study

**FIGURE 4:**
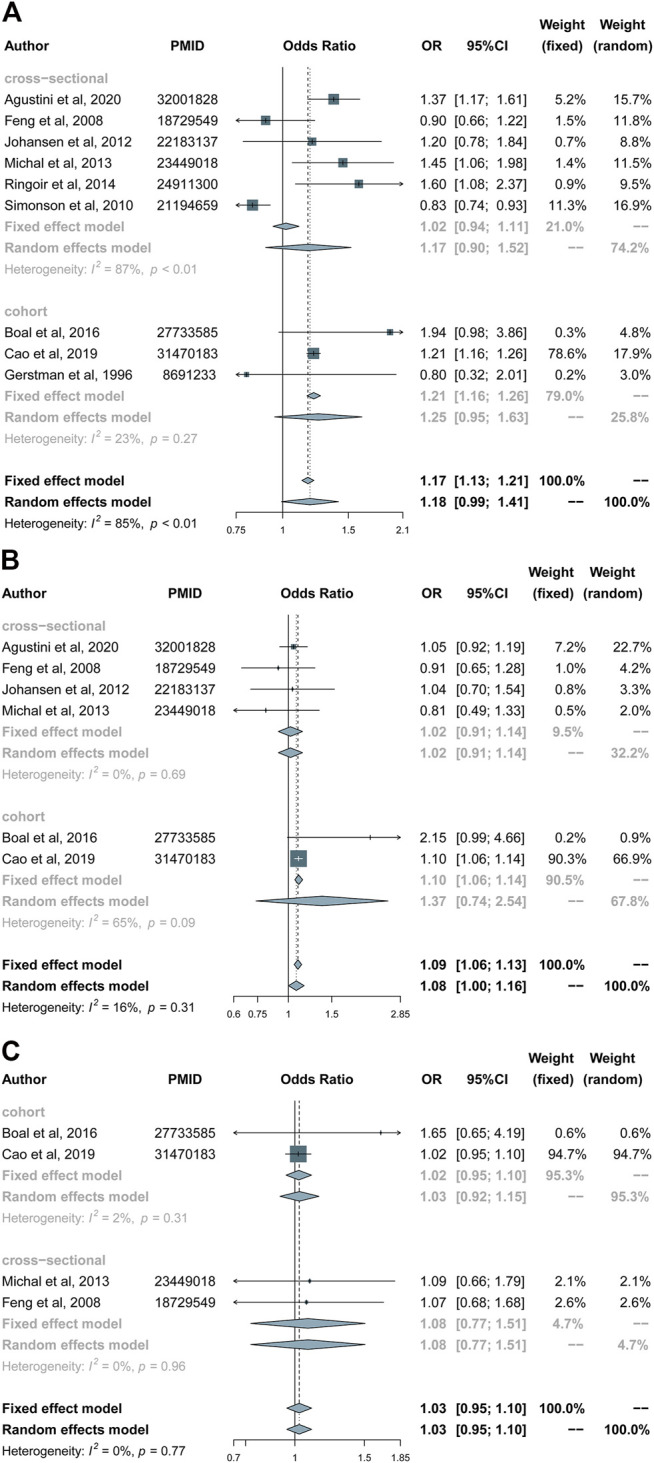
Forest plots of subgroup analysis according to the type of study (cohort/cross-sectional): **(A)** Beta blockers group. **(B)** Calcium channel blockers group. **(C)** Diuretics group.

Subgroup analysis on the basis of study type (cohort/cross-sectional) suggested that there was a significant connection for beta blockers (OR 1.21, 95% CI 1.16–1.26) usage in cohort studies and no significant connection between drug use and the risk of depression in cross-sectional studies (Figure 4).

### Network Meta-analysis

**FIGURE 5:**
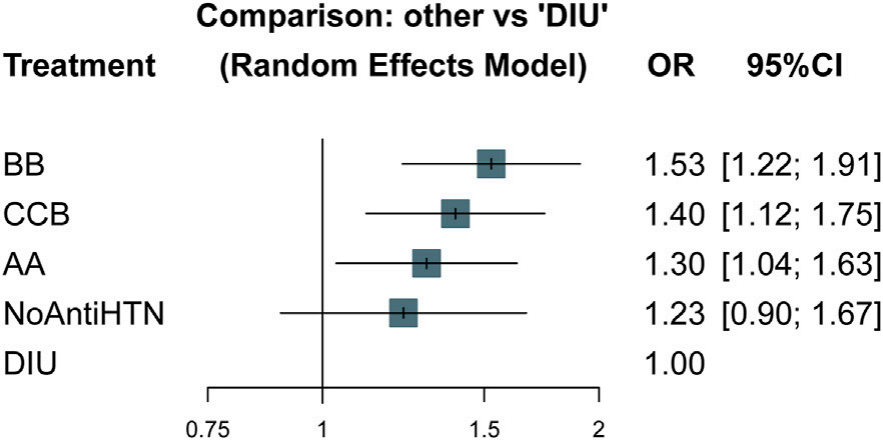
Forest plots of network meta-analysis: other vs. diuretics. OR, odds ratio; CI, confidence interval; AA, angiotensin antagonists; BB, beta blockers; CCB, calcium channel blockers; DIU, diuretics; NoAntiHTN, not taking antihypertensive medication.

A total of five studies, involving 263,025 participants, were included in the network meta-analysis. The network plot depicts the direct comparison between the treatment groups (Supplementary Figure S3). With reference to diuretics, except for the NoAntiHTN group, all antihypertensive treatments were significantly associated with higher risk of depression: beta blockers (OR 1.53, 95% CI 1.22–1.91), calcium channel blockers (OR 1.40, 95% CI 1.12–1.75) and angiotensin antagonists (OR 1.30, 95% CI 1.04–1.63) (Figure 5). Using NoAntiHTN group as comparison group, the odds ratios of network meta-analysis results were as follows: beta blockers 1.25 (95% CI 0.96–1.61), calcium channel blockers 1.14 (95% CI 0.88–1.48), angiotensin antagonists 1.06 (95% CI 0.81–1.39) and diuretics 0.82 (95% CI 0.60–1.11) (Supplementary Figure S4). The ranking P-score based on network meta-analysis was 0.056 for beta blockers, 0.305 for calcium channel blockers, 0.529 for angiotensin antagonists, 0.638 for NoAntiHTN, and 0.972 for diuretics (Supplementary Table S2).

There was moderate heterogeneity in the whole network meta-analysis (*p* = 0.011), and the between-designs Q statistic supported global consistency (*p* = 0.614). Local inconsistency in network meta-analysis is shown in Supplementary Table S3 and Supplementary Figure S5. The comparison-adjusted funnel plot analysis demonstrated no publication bias (Supplementary Figure S6). Taken together, these results demonstrate the reliability of the net meta-analysis.

## Discussion

To clarify the role of antihypertensives use in depression, we conducted a systematic review and network meta-analysis. Compared with diuretics, our network meta-analysis results suggested that beta blockers, calcium channel blockers or angiotensin antagonists usage promote depression.

The network meta-analysis does not prespecify the standard, and it only compares multiple treatments simultaneously in a single analysis. Hence, the OR and 95% CI were different when the reference was changed to NoAntiHTN, while the rank order remained constant. However, there were no significant differences among all comparisons versus NoAntiHTN, which may be due to the small sample sizes and low statistical power of the studies than that comparisons versus diuretics.

The results of our traditional meta-analyses and subgroup analysis indicated that only the calcium channel blockers and beta blockers may increase the risk of depression. The network meta-analysis overcomes the limitation in conventional meta-analysis, because it can compare multiple antihypertensive treatments at the same time, rather than being restricted to comparisons of one drug class versus all others (Elliott and Meyer, 2007). The effect of the angiotensin antagonists on depression, therefore, may have been underestimated. In addition, although traditional meta-analysis indicated that calcium channel blockers use was associated with increased risk of depression, it is worth noting that this conclusion to a large extent was dependent on the cohort study by Cao *et al.* Due to its large sample size and relatively long follow-up duration, this study accounted for 90% weight of the fixed effect model (Cao et al., 2019). This study may confound the association between depression and antihypertensives use. To clarify the elusive link between depression and antihypertensive medicine, larger sample-sized cohort studies are warranted. Notably, in subgroup meta-analysis, we found different results in cross-sectional and cohort studies, and cohort studies have advantages over cross-sectional studies. Differences between them may be related to study design, sample size, and duration of follow-up. The cross-sectional studies measure exposure and outcome simultaneously (Belbasis and Bellou, 2018), thus, only association can be established. As for cohort studies, as stated by Belbasis *et al.*, “a cohort study tracks two or more groups forward from exposure to outcome” (Belbasis and Bellou, 2018). Cohort studies have a clear temporality in support of causal inference. In our meta-analysis, number of included cohort studies was relatively small, so more articles with cohort design would be necessary to further verify our results.

Currently, most studies suggest that beta blockers usage is accompanied by increased susceptibility to depression. However, Simonson *et al.* reported a converse association between them. This study points out that the fact that the various findings are difficult to explain because of the diverse etiologies of the cardiovascular conditions (Simonson et al., 2011). Jeon *et al.* also found bidirectional association between blood pressure and symptoms of depression, in a large cohort study of young and middle-aged individuals (Jeon et al., 2020). A recent meta-analysis (Riemer et al., 2021) which investigated the risk of psychiatric adverse events during beta blockers therapy found no association between beta blockers use and depression, while the majority of beta blocker trials were conducted almost 20 years ago. The controversy regarding beta blockers use and depression may be explained by the heterogeneity of populations (Agustini et al., 2020). In the studies which found a positive or no association of beta blockers with symptoms of depression, the participants with a history of cardiovascular diseases, myocardial infarction or heart failure and distinct age groups were often included (Gerstman et al., 1996; Johansen et al., 2012), hence, the impact of beta blockers on general health improvement may outweigh or confound its effect on mood. It is therefore recommended that rigorous inclusion and exclusion criteria should be instituted in future studies focusing on the role of beta blockers in depression.

There are several limitations of this study that should be noted. First, there were moderate heterogeneity in network meta-analysis and considerable heterogeneity between studies in meta-analysis, even in the subgroup meta-analysis, which may influence the reliability of results. But, this is an inevitable problem. The type of study (cohort/cross-sectional) and measure of depression are both potential sources of heterogeneity. Indeed, there may be other unknown sources of heterogeneity. Therefore, the random-effects model was used to complete the network meta-analysis, which conservatively accounts for heterogeneity. Second, depression is approximately twice as prevalent in women as it is in men (Van de Velde et al., 2010). Yet much of the data from the studies we used were not stratified by gender. This prevented us from further assessing the differences between gender and risk of depression.

In conclusion, the outcome of this network meta-analysis supports the view that beta blockers, calcium channel blockers or angiotensin antagonists usage may be risk factors of depression. Our findings may be helpful in the management of depression by hypertensive individuals.

## Data Availability

The original contributions presented in the study are included in the article/Supplementary Material, further inquiries can be directed to the corresponding authors.
