# Supplementary material for: Antihypertensive Drug Use and the Risk of Depression: A Systematic Review and Network Meta-analysis
Source: Front Pharmacol. 2021 Nov 8;12:777987. doi: 10.3389/fphar.2021.777987 (PMC8606787; doi:10.3389/fphar.2021.777987)
Supplement: Supplementary file 1 [file DataSheet1.PDF]

Supplementary Material

Content:

Supplementary Materials:

Supplementary Appendix 1: Search terms for PubMed.

Supplementary Appendix 2: PRISMA 2020 Checklist.

Supplementary Appendix 3: The Agency for Healthcare Research and Quality for cross-sectional studies.

Supplementary Appendix 4: Newcastle – Ottawa Scale for cohort studies.

Supplementary Table S1: The scores of quality assessment for cross-sectional studies and cohort studies.

Supplementary Table S2: P-score in frequentist network meta-analysis.

Supplementary Table S3: Direct and indirect comparison in the network meta-analysis and local the inconsistency test results.

Supplementary Figure S1: Sensitivity analysis by detecting one study each time. **(A)**Angiotensin antagonists group. **(B)**Diuretics group. **(C)**Beta blockers group. **(D)**Calcium channel blockers group.

Supplementary Figure S2: Funnel plots for the assessment of publication bias in **(A)**beta blockers group, **(B)**angiotensin antagonists group, **(C)**calcium channel blockers group and **(D)**diuretics group.

Supplementary Figure S3: Network plot demonstrating direct evidence between the treatment groups.

Supplementary Figure S4: Forest plots of network meta-analysis: other vs. NoAntiHTN.

Supplementary Figure S5: Net heat plot of network meta-analysis.

Supplementary Figure S6: Comparison-adjusted funnel plot of network meta-analysis.

Supplementary Appendix 1: Search terms for PubMed.

Search (((((((antihypertensive drugs) OR (antihypertensive agents)) OR "Hypertension/drug effects"[Mesh]) OR "Hypertension/drug therapy"[Mesh]) OR "Blood Pressure/drug effects"[Mesh]) OR "Antihypertensive Agents"[Mesh]) OR "Antihypertensive Agents" [Pharmacological Action]) AND ((((((depressive symptoms) OR depression) OR depressed) OR depress) OR "Depression"[Mesh]) OR "Depressive disorder"[Mesh])

Supplementary Appendix 2: PRISMA 2020 Checklist.

Section and Topic	Item #	Checklist item	Location where item is reported
**TITLE**			
Title	1	Identify the report as a systematic review.	Page 1
**ABSTRACT**			
Abstract	2	See the PRISMA 2020 for Abstracts checklist.	Page 2
**INTRODUCTION**			
Rationale	3	Describe the rationale for the review in the context of existing knowledge.	Page 3
Objectives	4	Provide an explicit statement of the objective(s) or question(s) the review addresses.	Page 3
**METHODS**			
Eligibility criteria	5	Specify the inclusion and exclusion criteria for the review and how studies were grouped for the syntheses.	Page 3, 4
Information sources	6	Specify all databases, registers, websites, organisations, reference lists and other sources searched or consulted to identify studies. Specify the date when each source was last searched or consulted.	Page 3
Search strategy	7	Present the full search strategies for all databases, registers and websites, including any filters and limits used.	Page 3
Selection process	8	Specify the methods used to decide whether a study met the inclusion criteria of the review, including how many reviewers screened each record and each report retrieved, whether they worked independently, and if applicable, details of automation tools used in the process.	Page 3
Data collection process	9	Specify the methods used to collect data from reports, including how many reviewers collected data from each report, whether they worked independently, any processes for obtaining or confirming data from study investigators, and if applicable, details of automation tools used in the process.	Page 4
Data items	10a	List and define all outcomes for which data were sought. Specify whether all results that were compatible with each outcome domain in each study were sought (e.g. for all measures, time points, analyses), and if not, the methods used to decide which results to collect.	Page 4
	10b	List and define all other variables for which data were sought (e.g. participant and intervention characteristics, funding sources). Describe any assumptions made about any missing or unclear information.	Page 4
Study risk of bias assessment	11	Specify the methods used to assess risk of bias in the included studies, including details of the tool(s) used, how many reviewers assessed each study and whether they worked independently, and if applicable, details of automation tools used in the process.	Page 4
Effect measures	12	Specify for each outcome the effect measure(s) (e.g. risk ratio, mean difference) used in the synthesis or presentation of results.	Page 4
Synthesis methods	13a	Describe the processes used to decide which studies were eligible for each synthesis (e.g. tabulating the study intervention characteristics and comparing against the planned groups for each synthesis (item #5)).	Page 3
	13b	Describe any methods required to prepare the data for presentation or synthesis, such as handling of missing summary statistics, or data conversions.	Page 4, 5
	13c	Describe any methods used to tabulate or visually display results of individual studies and syntheses.	Page 4, 5
	13d	Describe any methods used to synthesize results and provide a rationale for the choice(s). If meta-analysis was performed, describe the model(s), method(s) to identify the presence and extent of statistical heterogeneity, and software package(s) used.	Page 4, 5
	13e	Describe any methods used to explore possible causes of heterogeneity among study results (e.g. subgroup analysis, meta-regression).	Page 4, 5
	13f	Describe any sensitivity analyses conducted to assess robustness of the synthesized results.	Page 4
Reporting bias	14	Describe any methods used to assess risk of bias due to missing results in a synthesis (arising from reporting biases).	Page 4

Section and Topic	Item #	Checklist item	Location where item is reported
assessment			
Certainty assessment	15	Describe any methods used to assess certainty (or confidence) in the body of evidence for an outcome.	Page 4, 5
**RESULTS**			
Study selection	16a	Describe the results of the search and selection process, from the number of records identified in the search to the number of studies included in the review, ideally using a flow diagram.	Page 5
	16b	Cite studies that might appear to meet the inclusion criteria, but which were excluded, and explain why they were excluded.	Not applicable
Study characteristics	17	Cite each included study and present its characteristics.	Page 5, 13, 14
Risk of bias in studies	18	Present assessments of risk of bias for each included study.	Page 4
Results of individual studies	19	For all outcomes, present, for each study: (a) summary statistics for each group (where appropriate) and (b) an effect estimate and its precision (e.g. confidence/credible interval), ideally using structured tables or plots.	Page 5, 6, 7
Results of syntheses	20a	For each synthesis, briefly summarise the characteristics and risk of bias among contributing studies.	Page 5, 6, 7
	20b	Present results of all statistical syntheses conducted. If meta-analysis was done, present for each the summary estimate and its precision (e.g. confidence/credible interval) and measures of statistical heterogeneity. If comparing groups, describe the direction of the effect.	Page 5, 6, 7
	20c	Present results of all investigations of possible causes of heterogeneity among study results.	Page 6
	20d	Present results of all sensitivity analyses conducted to assess the robustness of the synthesized results.	Page 5, 6
Reporting biases	21	Present assessments of risk of bias due to missing results (arising from reporting biases) for each synthesis assessed.	Page 5, 6
Certainty of evidence	22	Present assessments of certainty (or confidence) in the body of evidence for each outcome assessed.	Page 6, 7
**DISCUSSION**			
Discussion	23a	Provide a general interpretation of the results in the context of other evidence.	Page 7
	23b	Discuss any limitations of the evidence included in the review.	Page 8
	23c	Discuss any limitations of the review processes used.	Page 8
	23d	Discuss implications of the results for practice, policy, and future research.	Page 8
**OTHER INFORMATION**			
Registration and protocol	24a	Provide registration information for the review, including register name and registration number, or state that the review was not registered.	Registration does not apply.
	24b	Indicate where the review protocol can be accessed, or state that a protocol was not prepared.	Same as above
	24c	Describe and explain any amendments to information provided at registration or in the protocol.	Same as above
Support	25	Describe sources of financial or non-financial support for the review, and the role of the funders or sponsors in the review.	Page 9

Section and Topic	Item #	Checklist item	Location where item is reported
Competing interests	26	Declare any competing interests of review authors.	Page 8
Availability of data, code and other materials	27	Report which of the following are publicly available and where they can be found: template data collection forms; data extracted from included studies; data used for all analyses; analytic code; any other materials used in the review.	Not applicable

From: Page MJ, McKenzie JE, Bossuyt PM, Boutron I, Hoffmann TC, Mulrow CD, et al. The PRISMA 2020 statement: an updated guideline for reporting systematic reviews. BMJ 2021;372:n71. doi: 10.1136/bmj.n71

For more information, visit: http://www.prisma-statement.org/

Supplementary Appendix 3: The Agency for Healthcare Research and Quality for cross-sectional studies

Item	Yes	No	Unclear
1) Define the source of information (survey, record review)			
2) List inclusion and exclusion criteria for exposed and unexposed subjects (cases and controls) or refer to previous publications			
3) Indicate time period used for identifying patients			
4) Indicate whether or not subjects were consecutive if not population-based			
5) Indicate if evaluators of subjective components of study were masked to other aspects of the status of the participants			
6) Describe any assessments undertaken for quality assurance purposes (e.g., test/retest of primary outcome measurements)			
7) Explain any patient exclusions from analysis			
8) Describe how confounding was assessed and/or controlled.			
9) If applicable, explain how missing data were handled in the analysis			
10) Summarize patient response rates and completeness of data collection			
11) Clarify what follow-up, if any, was expected and the percentage of patients for which incomplete data or follow-up was obtained			

Supplementary Appendix 4: Newcastle – Ottawa Scale for cohort studies

SELECTION

1) Representativeness of the Exposed Cohort

Item is assessing the representativeness of exposed individuals in the community, not the representativeness of the sample of women from some general population. For example, subjects derived from groups likely to contain middle class, better educated, health oriented women are likely to be representative of postmenopausal estrogen users while they are not representative of all women (e.g. members of a health maintenance organisation (HMO) will be a representative sample of estrogen users. While the HMO may have an under-representation of ethnic groups, the poor, and poorly educated, these excluded groups are not the predominant users users of estrogen).

Allocation of stars as per rating sheet

2) Selection of the Non-Exposed Cohort

Allocation of stars as per rating sheet

3) Ascertainment of Exposure

Allocation of stars as per rating sheet

4) Demonstration That Outcome of Interest Was Not Present at Start of Study

In the case of mortality studies, outcome of interest is still the presence of a disease/ incident, rather than death. That is to say that a statement of no history of disease or incident earns a star.

COMPARABILITY

1) Comparability of Cohorts on the Basis of the Design or Analysis

A maximum of 2 stars can be allotted in this category
Either exposed and non-exposed individuals must be matched in the design and/or confounders must be adjusted for in the analysis. Statements of no differences between groups or that differences were not statistically significant are not sufficient for establishing comparability. Note: If the relative risk for the exposure of interest is adjusted for the confounders listed, then the groups will be considered to be comparable on each variable used in the adjustment.
There may be multiple ratings for this item for different categories of exposure (e.g. ever vs. never, current vs. previous or never)
Age = ☆, Other controlled factors = ☆

OUTCOME

1) Assessment of Outcome

For some outcomes (e.g. fractured hip), reference to the medical record is sufficient to satisfy the requirement for confirmation of the fracture. This would not be adequate for vertebral fracture outcomes where reference to x-rays would be required.

a) Independent or blind assessment stated in the paper, or confirmation of the outcome by reference to secure records (x-rays, medical records, etc.)☆
b) Record linkage (e.g. identified through ICD codes on database records) ☆
c) Self-report (i.e. no reference to original medical records or x-rays to confirm the outcome)
d) No description.

2) Was Follow-Up Long Enough for Outcomes to Occur

An acceptable length of time should be decided before quality assessment begins (e.g. 5 yrs. for exposure to breast implants)

3) Adequacy of Follow Up of Cohorts

This item assesses the follow-up of the exposed and non-exposed cohorts to ensure that losses are not related to either the exposure or the outcome.

Allocation of stars as per rating sheet

Supplementary Table S1: The scores of quality assessment for cross-sectional studies and cohort studies

Author	Study type	Sample	NOS	AHRQ
Agustini *et al*	cross-sectional	14195		6
Boal *et al*	cohort	144066	8	
Cao *et al*	cohort	181709	8	
Feng *et al*	cross-sectional	2804		7
Gerstman *et al*	cohort	3782	7	
Johansen *et al*	cohort	55472		7
Michal *el al*	cross-sectional	5000		8
Ringoir *et al*	cross-sectional	573		6
Simonson *et al*	cross-sectional	7272		5

NOS, Newcastle – Ottawa Scale; AHRQ, The Agency for Healthcare Research and Quality.

Supplementary Table S2: P-score in frequentist network meta-analysis.

**Drug**	**P-score (random)**
DIU	0.972
NoAntiHTN	0.638
AA	0.529
CCB	0.305
BB	0.056

DIU, diuretics; NoAntiHTN, no antihypertensive medication treatment; AA, angiotensin antagonists; CCB, calcium channel blockers; BB, beta blockers.

Supplementary Table S3: Direct and indirect comparison in the network meta-analysis and local the inconsistency test results

Comparison	No. of Studies	Prop	NMA	Direct	Indirect	*p*-value
AA: BB	5	0.98	0.86	0.86	1.12	0.140
AA: CCB	5	0.99	0.93	0.94	0.58	0.038
AA: DIU	3	0.97	1.41	1.44	0.69	0.025
AA: NoAntiHTN	2	0.21	1.08	0.65	1.23	0.009
BB: CCB	5	1.00	1.08	1.08	0.41	0.109
BB: DIU	3	0.99	1.64	1.62	4.96	0.038
BB: NoAntiHTN	2	0.46	1.25	1.31	1.20	0.653
CCB: DIU	3	0.98	1.51	1.53	0.74	0.091
CCB: NoAntiHTN	2	0.42	1.16	1.32	1.05	0.249
DIU: NoAntiHTN	1	0.08	0.77	1.06	0.74	0.379

Prop, direct evidence proportion; NMA, estimated treatment effect (OR) in network meta-analysis; Direct, estimated treatment effect (OR) derived from direct evidence; Indirect, estimated treatment effect (OR) derived from indirect evidence; AA, angiotensin antagonists; BB, beta blockers; CCB, calcium channel blockers; DIU, diuretics; NoAntiHTN, no antihypertensive medication treatment.

Supplementary Figures

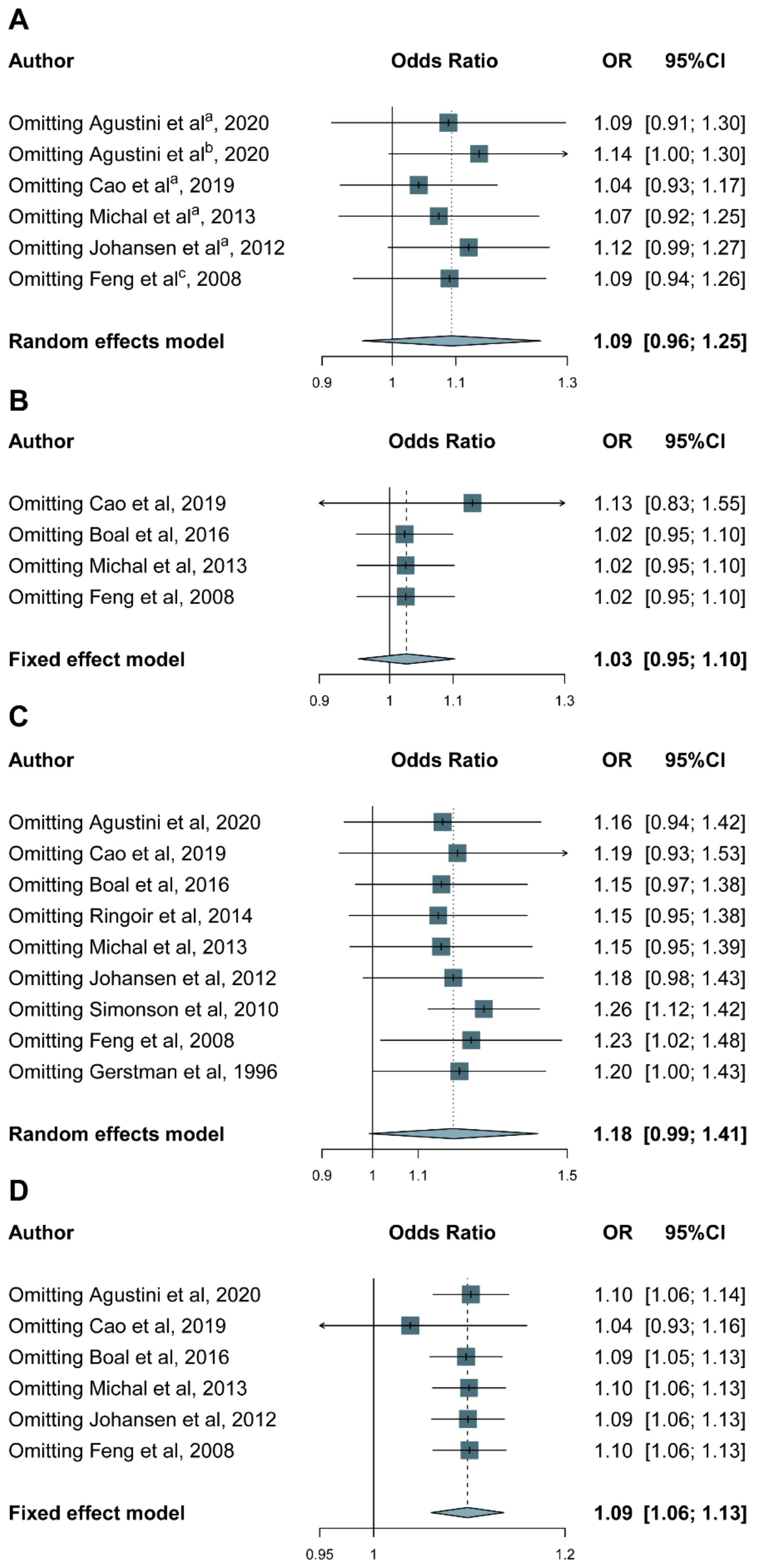

Supplementary Figure S1: Sensitivity analysis by detecting one study each time. **(A)**Angiotensin antagonists group. **(B)**Diuretics group. **(C)**Beta blockers group. **(D)**Calcium channel blockers group. [a, b] and [c] indicate angiotensin-converting enzyme inhibitors, angiotensin receptor blockers and angiotensin antagonists, respectively.

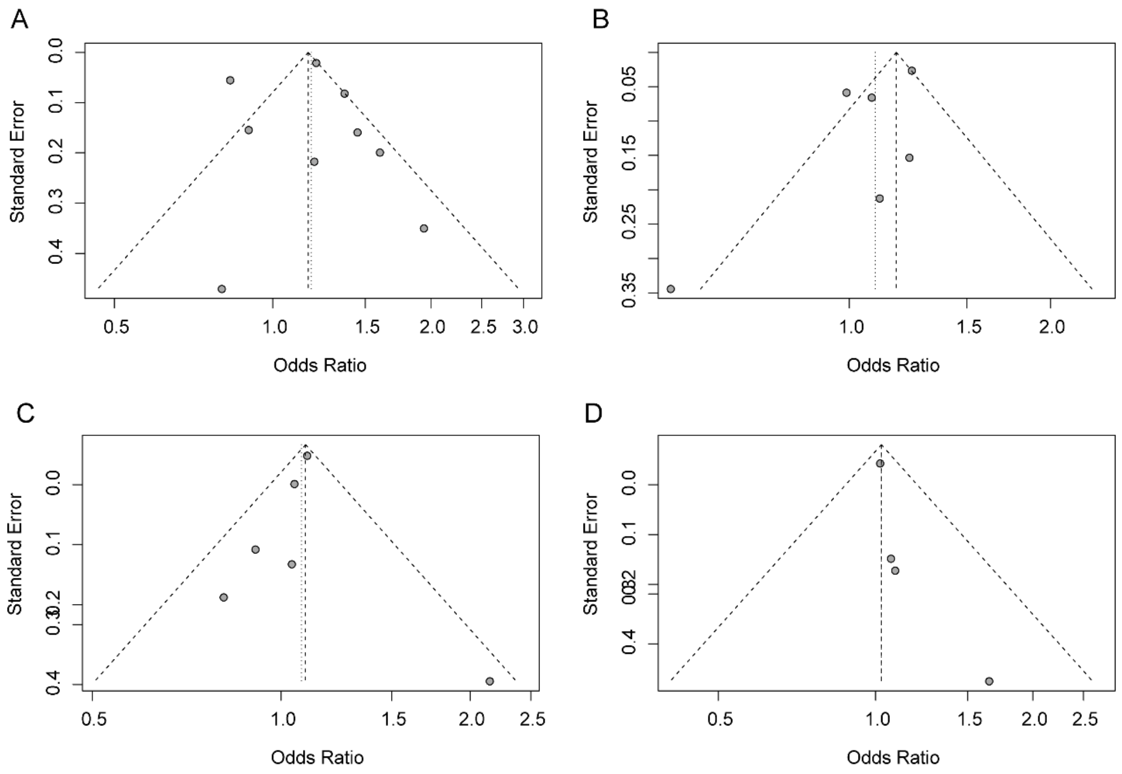

Supplementary Figure S2: Funnel plots for the assessment of publication bias in **(A)**beta blockers group, **(B)**angiotensin antagonists group, **(C)**calcium channel blockers group and **(D)**diuretics group.

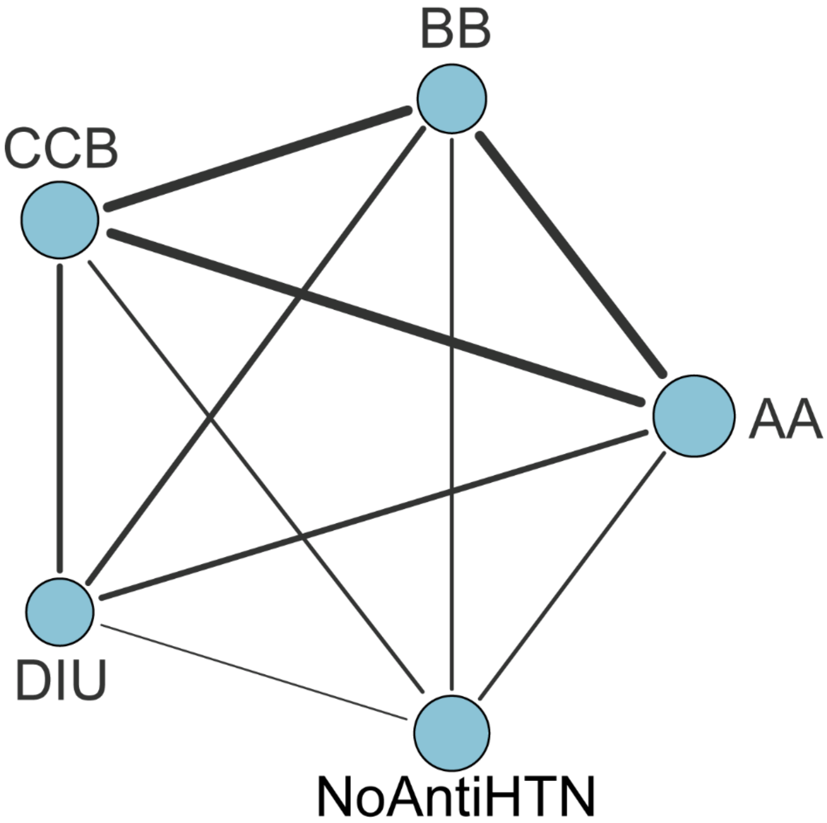

Supplementary Figure S3: Network plot demonstrating direct evidence between the treatment groups. AA, angiotensin antagonists; BB, beta blockers; CCB, calcium channel blockers; DIU, diuretics; NoAntiHTN, no antihypertensive medication treatment.

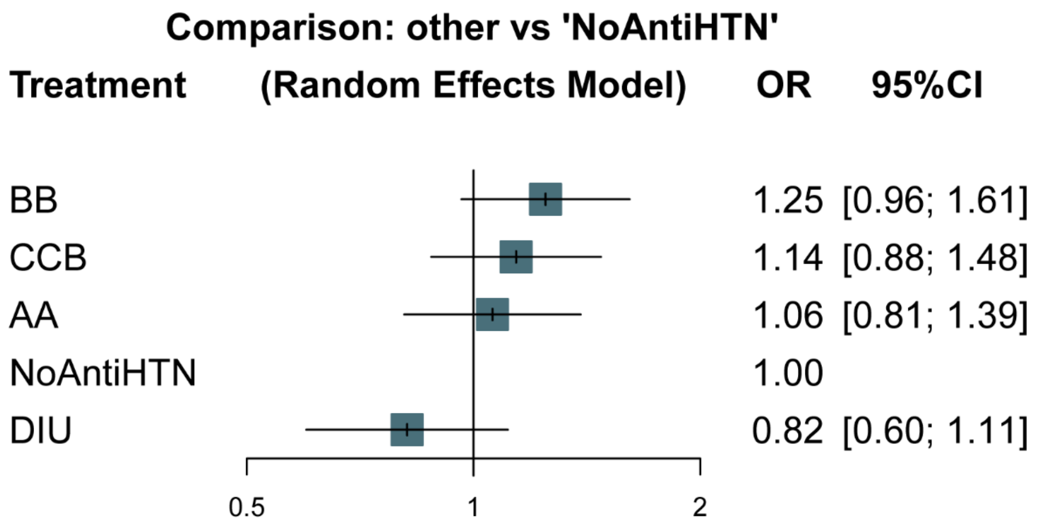

Supplementary Figure S4: Forest plots of network meta-analysis: other vs. NoAntiHTN. OR, odds ratio; CI, confidence interval; AA, angiotensin antagonists; BB, beta blockers; CCB, calcium channel blockers; DIU, diuretics; NoAntiHTN, not taking antihypertensive medication.

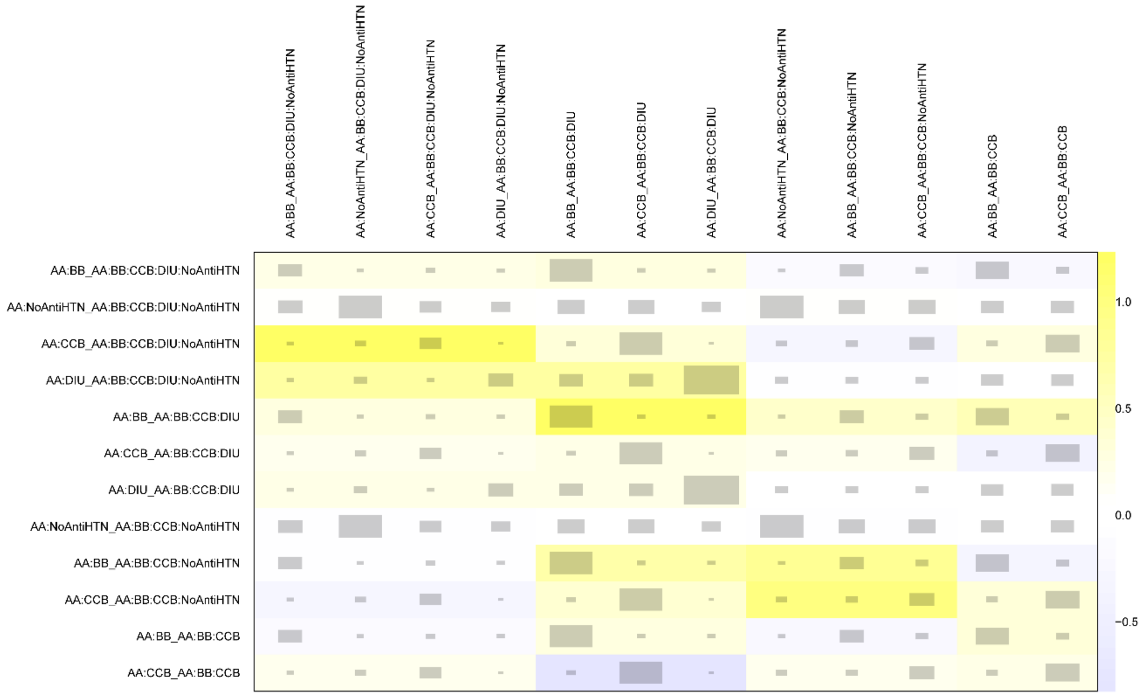

Supplementary Figure S5: Net heat plot of network meta-analysis.

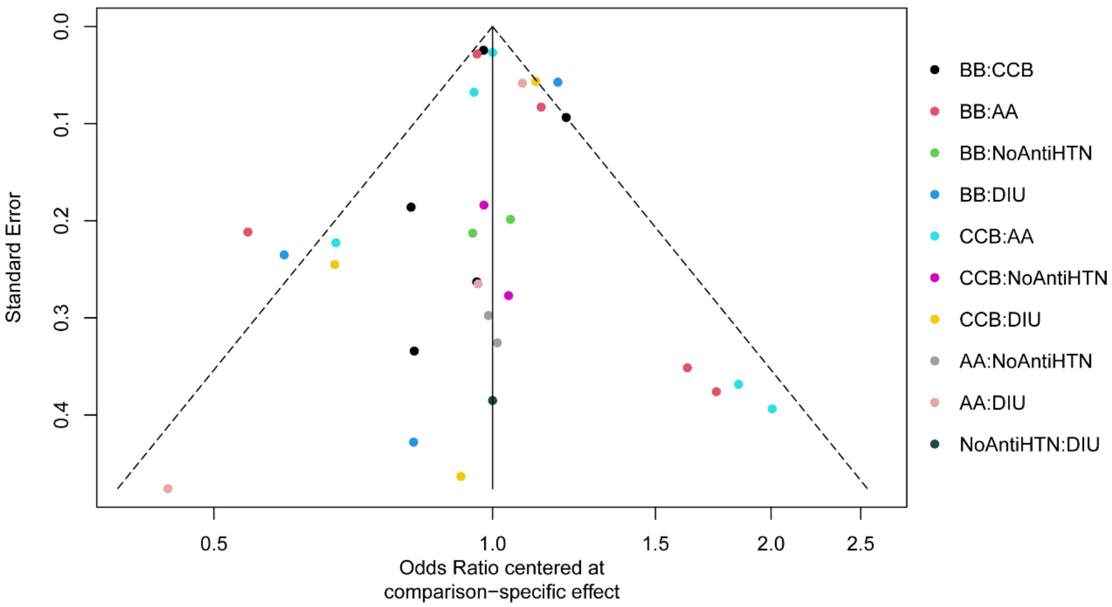

Supplementary Figure S6: Comparison-adjusted funnel plot of network meta-analysis. AA, angiotensin antagonists; BB, beta blockers; CCB, calcium channel blockers; DIU, diuretics; NoAntiHTN, not taking antihypertensive medication.
